# Resting-state fMRI markers of conversion to dementia in amnestic MCI: a pilot study

**DOI:** 10.1192/j.eurpsy.2023.418

**Published:** 2023-07-19

**Authors:** E. Abdullina, Y. Panikratova, E. Ponomareva, I. Roshchina, N. Selezneva, I. Lebedeva, S. Gavrilova

**Affiliations:** Mental Health Research Center, Moscow, Russian Federation

## Abstract

**Introduction:**

Patients with amnestic mild cognitive impairment (aMCI) have a high risk of transition to Alzheimer’s disease. Analysis of potential biomarkers of conversion to dementia in this clinical group is crucial for prognosis and early intervention.

**Objectives:**

The aim of the pilot study was to compare whole-brain functioning characteristics (fMRI, spontaneous activity and local coherence) in aMCI converters and non-converters to dementia.

**Methods:**

Nine aMCI converters to dementia of the Alzheimer’s type (mean age 69.2 ± 8.2; 9 females) and ten aMCI non-converters (mean age 65.9 ± 6.1; 8 females) underwent resting-state fMRI (3T). All patients were followed up for three years. Baseline whole-brain amplitude of low-frequency fluctuations (ALFF) and local coherence (LCOR) were compared between groups (CONN-fMRI toolbox 19.с, https://web.conn-toolbox.org/; *p* < .001 voxelwise, *p(FDR)* < .05 clusterwise). Age was included in the analyses as a second-level covariate.

**Results:**

As compared to non-converters, aMCI converters were characterized by higher ALFF and LCOR values in the cluster located in the frontal medial cortex and frontal pole bilaterally.

**Conclusions:**

Frontal medial cortex and frontal pole are involved in a wide range of cognitive functions, including episodic memory and “hot” (motivational) executive control (Rolls. ProgNeurobiol 2022; 217; Friedman, Robbins. Neuropsychopharmacology 2022; 47(1) 72-89). Both increased and decreased LCOR/ALFF values in aMCI converters compared to non-converters were found, although in the other regions (Mondragón et al. Dement Geriatr Cogn Dis Extra 2021; 11(3) 235–249; Khatri, Kwon. Front Aging Neurosci. 2022; 14). It seems reasonable to clarify if the brain functional features revealed in our study are the markers of conversion to dementia in aMCI.

**Disclosure of Interest:**

None Declared

